# Disrespect and abuse of women during childbirth at health facilities in Eastern Africa: systematic review and meta-analysis

**DOI:** 10.3389/fmed.2023.1117116

**Published:** 2023-04-20

**Authors:** Natnael Atnafu Gebeyehu, Gtachew Asmare Adella, Kirubel Dagnaw Tegegne

**Affiliations:** ^1^School of Midwifery, College of Health Science and Medicine, Wolaita Sodo University, Wolaita Sodo, Ethiopia; ^2^School of Public Health, College of Health Sciences and Medicine, Wolaita Sodo University, Wolaita Sodo, Ethiopia; ^3^Department of Nursing, College of Medicine and Health Sciences, Wollo University, Dessie, Ethiopia

**Keywords:** disrespect, abuse, mistreatment, childbirth, meta-analysis, East Africa

## Abstract

**Background:**

Disrespectful and abusive maternity care is a sign of poor treatment that influences women’s choice to deliver their babies in institutions. Such malpractices continue to go unreported and are rarely exposed in developing countries, despite their serious burden. Therefore, this meta-analysis study aimed to estimate disrespect and abuse of women during childbirth in East Africa.

**Methods:**

PubMed, Google Scholar, Scopus, and Science Direct databases were searched. Data were extracted using Microsoft Excel and analyzed using STATA statistical software (v. 14). Publication bias was checked by forest plot, Begg’s rank test, and Egger’s regression test. To look for heterogeneity, I^2^ was computed, and an overall estimated analysis was carried out. Subgroup analysis was done by study region, sample size, and publication. The pooled odds ratio for associated factors was also computed.

**Results:**

Out of 654 articles assessed, 18 met the criteria and were included in this study. There were a total of 12,434 study participants. The pooled prevalence of disrespect and abuse of women during childbirth in East Africa was 46.85% (95% CI: 45.26.72–66.98), I^2^ = 81.9%. It was lower in studies with sample size greater than 5000 (33%). The disrespect and abuse rates between community-based studies (44.96%) and institutional-based studies (47.35%) did not differ significantly, though. Instrumental delivery (AOR = 2.70; 95%CI: 1.79–4.08), presence of complications (AOR = 6.41; 95% CI: 1.36–30.14), receiving care at government hospitals (AOR = 3.66; 95% CI: 1.09–12.23), and poor wealth index (AOR = 2.16; 95% CI: 1.26–3.70) were associated factors.

**Conclusion:**

In East Africa, disrespect and abuse of women during childbirth was high. Instrumental delivery, presence of complications during childbirth, receiving care at government hospitals and poor wealth index were predictors of maternal disrespect and abuse. Safe delivery practice should be promoted. Training in compassionate and respectful maternity care, particularly in public hospitals, has also been recommended.

## Introduction

Maternal mortality and morbidity among women of reproductive age in poor nations are primarily caused by complications during pregnancy and childbirth (1). There is widespread agreement that professional birthing services provided within a formal healthcare system can reduce the risk of maternal fatalities (2). More than 162,000 women still lose their lives in pregnancy and childbirth each year in sub-Saharan Africa (SSA). Most such fatalities occur within a day of delivery (3).

The East African countries are Burundi, Ethiopia, Comoros, Uganda, Rwanda, Tanzania, Mozambique, Madagascar, Zimbabwe, Kenya, and Zambia are among the poorest in the world and have low access to and affordability of maternal healthcare services (4). Moreover, the risk of adverse effects on the mother and the child is increased by a lack of access to appropriate obstetric care, particularly during delivery. Due to this service gap, women end up having poor maternal outcomes. In East Africa, the risk of death for those mothers is 1 in 31, compared to 1 in 4,300 in a high-income country (5).

A woman’s rights during maternity care are violated by disrespect and abuse (D&A), which is also referred to as mistreatment, obstetric violence, or dehumanized care (6). Women’s human rights are violated when they are treated disrespectfully and violently while receiving facility-based maternity care. This also keeps women from using maternal health services, erodes their satisfaction and confidence in the healthcare system, and results in unfavorable pregnancy outcomes (7–10). Respectful maternal care during facility-based maternity care is recommended by the World Health Organization (WHO) as a fundamental method for improving the quality of maternity services, to decrease disrespect and abuse as well as maternal mortality and morbidity (11). The biggest obstacle to receiving quality maternity care globally, however, is the growing evidence of the disrespect, abuse, and mistreatment of women throughout labor, delivery, and the postpartum period (10, 12, 13).

Growing data demonstrates that disrespect and abuse of maternal care, to varied degrees and severity, happens globally (14). Additionally, this research demonstrates that women get disrespectful and harsh care during childbirth (14). To ensure physical safety during institutional delivery, the quality of maternal health services is constrained (15, 16). Many women, however, do not receive the medically and culturally appropriate treatment that they require (17).

According to the Universal Declaration of the Rights of the Childbearing Woman, every woman has the right to sexual and reproductive healthcare that is respectful and dignified, including during childbirth (18, 19). As a result, abuse during childbirth may constitute a violation of women’s fundamental human rights (20) and may act as a strong deterrent to women seeking care in facilities for subsequent pregnancies (21–23). A seven-category model was proposed by Bowser and Hill in a landscape analysis of the evidence for disrespect and abuse in facility-based childbirth that was published (24). This model was intended to generate discussion and a research agenda for implementation, not to provide a thorough analysis of the available evidence. Physical abuse, non-consented clinical care, non-confidential care, substandard care, discrimination, abandonment, and imprisonment in health facilities (23) have all been major themes in more recent research on this subject (25–28).

Stress, exhaustion, irritation, and a lack of job satisfaction all have an impact on a healthcare provider’s negative behavior. The working environment and conditions at the facility, as well as work-related issues like severe workloads, long working hours, lax supervision, strained relationships with coworkers, and inadequate pay, all have an impact on these problems (7, 24, 29). Disrespect and abusive at these facilities has reportedly been linked to women’s underuse of healthcare facilities during birthing (23, 30, 31).

Several negative effects of D&A on women’s health and wellbeing have been documented, including fear about using medical facilities (24), an increased chance of birth complications (32), low self-ratings of health, sleeping issues, and symptoms of post-traumatic stress disorder (33). The prevalence of disrespect and abuse ranged from 15% in Tanzania (28) to 98% in Nigeria (27).

There are no data at the country level, despite the fact that numerous primary researches have confirmed disrespect and abuse during child birth in East Africa. Therefore, the goal of this systematic review and meta-analysis study was to determine the prevalence of disrespect and abuse of women during childbirth in East Africa and its determinants. Clinicians and other stakeholders will be able to address gaps in disrespect and abuse of women during childbirth and operational plans based on the study’s findings, which will provide them with the fundamental knowledge they need to provide every child bearing women.

## Methods

### Data synthesis and reporting

We conducted data analysis based on a single measurement result (disrespect and abuse). Tables, text, and a forest plot are used to present the results. Using the standard PRISMA checklist guideline, this systematic review and meta-analysis study was carried out to assess the overall prevalence of disrespect and abuse of women during child birth in East Africa (34) (Supplementary Table 1).

### Search strategy

Articles on the disrespect and abuse in East Africa were searched using international online databases (Pub Med, Science Direct, Scopus, EMBASE, and Google Scholar). The following keywords and search terms were used during the search: “prevalence,” “disrespect,” “abuse,” “delivery,” “obstetric,” “parturition,” “maternity care,” “mistreatment during pregnancy,” “attitude of health personnel,” “professional misconduct,” and “East Africa.” Boolean operators like “OR” and “AND” were used to combine the search phrases as well as use them separately. The Population Exposure Controls and Outcome (PECO) searching guidelines were used to perform the search strategy and retrieve relevant articles from the databases specified above. The search was carried out from October 1, 2022, until November 1, 2022.

### Study outcome

Any act of physical abuse, non-confidential care, non-consented care, non-dignified care, abandonment of care, discrimination, or detention in the facilities during childbirth is considered disrespectful and abusive behavior (35).

### Inclusion and exclusion criteria

The papers that were included in this meta-analysis were those that were conducted in East African nations, were published in English, and had full texts that could be searched. Studies that included data on the disrespect and abuse of child birth were also reported on. Qualitative studies, studies from developed countries, research from duplicated sources, and articles missing the complete text were all omitted from this systematic review and meta-analysis. The eligibility of the included articles in this study was determined using the COCOPOP (Condition, Context, and Population) paradigm. Laboring women made up the study population (POP), with the prevalence of disrespect and abuse serving as the condition (CO), and only studies carried out in East-Africa serving as the context (CO).

### Quality assessment

Two authors (NG and KT) independently assessed the standard of the research using the Joanna Briggs Institute (JBI) standardized quality appraisal checklist (36). The disagreement raised during the quality assessment was resolved through a discussion led by the third author (GA). The critical analysis checklist has eight parameters with yes, no, unclear, and not applicable options. The parameters involve the following questions:

(1) Where were the criteria for inclusion in the sample clearly defined?
(2) Were the study subjects and, therefore, the setting described in detail?
(3) Was the exposure measured result validly and reliably?
(4) Were the main objective and standard criteria used to measure the event?
(5) Were confounding factors identified?
(6) Were strategies to affect confounding factors stated?
(7) Were the results measured indeed and dependably? And (8) Was the statistical analysis suitable? Studies were considered low risk when they scored 50% and above on the quality assessment indicators, as reported in a Supplementary file (Supplementary Table 2).

### Risk of bias assessment

Through the Hoy et al. (37) established bias assessment tool, which consists of 10 items that assess four domains of bias as well as internal and external validity, two writers (NG and GA) independently evaluated included papers for risk of bias. Any disagreement raised during the risk of bias assessment was resolved through a discussion led by the third author (KT). Finally, the argument was solved and reached with an agreement. The first four questions (items 1–4) assess whether there is selection bias, non–response bias, and external validity. The last six questions (items 5–10) evaluate the presence of measurement bias, analysis-related bias, and internal validity. Studies were labeled as having a “low risk of bias” if they answered “yes” to eight or more of the ten questions. Studies classed as “moderate risk” if they answered “yes” to six to seven of the ten questions, whereas studies classified as “high risk” if they answered “yes” to five or fewer of the ten questions, according to information in a Supplementary file (Supplementary Table 3).

### Data extraction

Data extraction and analysis were carried out using STATA 14 software and a Microsoft Excel spreadsheet from 2016, respectively. Using a standardized Joanna Briggs Institute data extraction format, two authors (NG and KT) separately extracted all pertinent data. The disagreement raised during data extraction was resolved through a discussion led by the third author (GA). Finally, the argument was solved and reached with an agreement. Due to this study’s lack of a paper form, the data automation tool was not employed (manual data). The first author’s name, the year of publication, the study region, the study setting, the study design, the sample size, the prevalence of the prevalence of disrespect and abuse, the unadjusted odd ratio for variables, and the quality of each paper were retrieved.

### Data analysis

The data were exported to STATA software version 14 for analysis after being extracted from all pertinent findings in a Microsoft Excel spreadsheet. A weighted inverse variance random-effects model was used in a meta-analysis to produce a pooled OR. The presence of heterogeneity was visually evaluated using a forest plot, which was then utilized to analyze and estimate the pooled estimate of disrespect and abuse. Analysis of subgroups was done based on the study setting and sample size. Sensitivity analysis was used to determine the impact of a single study on the meta-analysis estimate of prevalence as a whole. The funnel plot was used to examine potential publication bias, and Begg and Egger’s regression tests were used to examine it more objectively. The trim-and-fill method proposed by Duval and Tweedie (38) was used to control publication bias. Cochran’s Q X^2^ test and I^2^ statistics were used to test for heterogeneity, estimate the amount of total/residual heterogeneity, and measure variability caused by heterogeneity, respectively (39). A Univar ate meta-regression analysis was used to examine the effects of sample size and publication year variations on between-study heterogeneity (40).

## Results

### Search results and study characteristics

**FIGURE 1:**
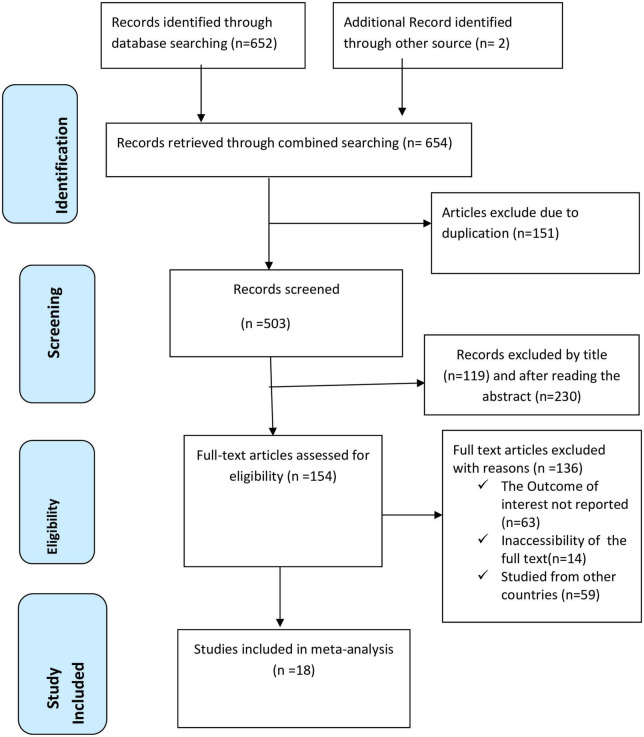
PRISMA flow chart displays the article selection process for the prevalence of disrespect and abuse of women during childbirth in East Africa.

We retrieved a total of 654 articles from various international online databases, such as PubMed, Scopus, EMBASE, Science Direct, and Google Scholar. After excluding duplicate research, we were left with 503 studies that were chosen for full title and abstract screening. The remaining 154 studies were screened for full text articles after 349 studies were eliminated because of the titles and abstracts. After reviewing the full text, 136 articles were excluded for further reasons. Finally, this systematic review and meta-analysis study’s inclusion criteria included 18 articles (26, 28, 35, 41–56) with 12,434 study participants (Figure 1).

Each of the included studies yielded low risk results and used a cross-sectional study methodology. The remaining studies were community-based, while 14 were cross-sectional studies carried out at healthcare facilities. Thirteen studies were carried out in Ethiopia (41, 43–48, 50–56), three in Tanzania (26, 28, 42), one in Kenya (49), and one in Sudan (51). Sample sizes varied from 204 to 2109. Disrespect and abuse of women during childbirth varied in prevalence from 1.9 to 98.9% (Table 1).

**TABLE 1:** Characteristics of studies included in the systematic review and meta-analysis of disrespect and abuse of women during childbirth in East Africa.

References	Country	Setting	Design	Sample size	Mean age	Prevalence	Response rate	Quality
Mihret et al. (41)	Ethiopia	Institutional	Cross-sectional	369	NR	15.9%	98.6%	Low-risk
Phillipo (42)	Tanzania	Institutional	Cross-sectional	263	NR	31.2%	100%	Low-risk
Samdo et al. (12)	Tanzania	Institutional	Cross-sectional	1914	25	15%	NR	Low-risk
Altahir et al. (51)	South Sudan	Institutional	Cross-sectional	2109	28	77.2%%	100%	Low-risk
Siraji et al. (43)	Ethiopia	Institutional	Cross-sectional	290	NR	91.7%	100%	Low-risk
Mengistie Zeleke and Melkie Bayeh (44)	Ethiopia	Institutional	Cross-sectional	407	29.11	49.6%	97.1%	Low-risk
Mekonnen et al. (45)	Ethiopia	Institutional	Cross-sectional	565	25.2	37.5%	97.4%	Low-risk
Kruk et al. (26)	Tanzania	Community	Cross-sectional	1779	25.86	19.48%	70.6%	Low-risk
Maldie et al. (46)	Ethiopia	Institutional	Cross-sectional	374	30.05	79.4%	98.7%	Low-risk
Gebremichael et al. (47)	Ethiopia	Community	Cross-sectional	1124	26.8	22%	100%	Low-risk
Banks et al. (48)	Ethiopia	Institutional	Cross-sectional	204	NR	21.1%	100%	Low-risk
Makumi et al. (49)	Kenya	Community	Cross-sectional	207	NR	71.9%	87.3%	Low-risk
Wassihun et al. (50)	Ethiopia	Community	Cross-sectional	422	28.6	67.1%	97.2%	Low-risk
Ukke et al. (52)	Ethiopia	Institutional	Cross-sectional	281	28.5	98.9%	100%	Low-risk
Kebede et al. (53)	Ethiopia	Institutional	Cross-sectional	409	31.3	52.1%	100%	Low-risk
Tagesse et al. (54)	Ethiopia	Institutional	Cross-sectional	557	26.8	46.9	95%	Low-risk
Negash et al. (55)	Ethiopia	Institutional	Cross-sectional	548	26	46.9%	95%	Low-risk
Tekle Bobo et al. (56)	Ethiopia	Institutional	Cross-sectional	612	NR	74.8%	100	Low-risk

## Meta-analysis

### Prevalence of disrespect and abuse during childbirth

**FIGURE 2:**
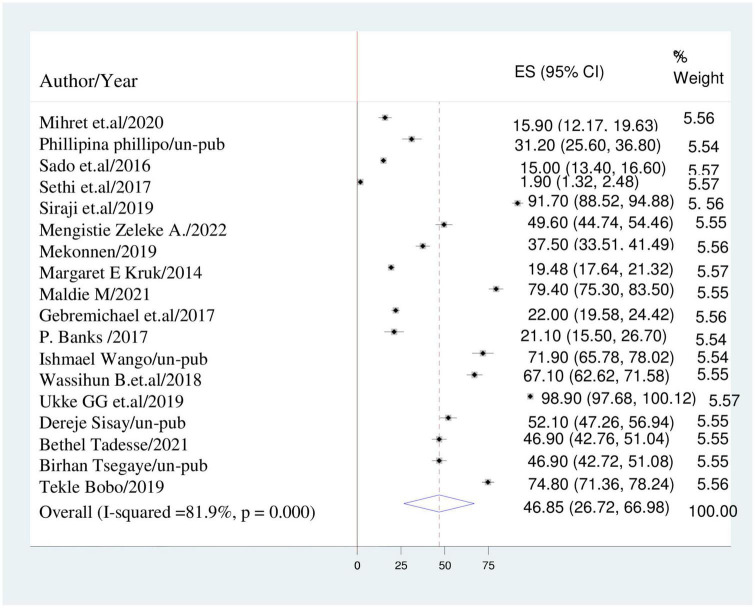
Forest plot displaying the pooled prevalence of disrespect and abuse of women during childbirth in East-Africa.

The overall estimate of disrespect and abuse during childbirth was computed using a DerSimonian and Laird random-effects model. As a result, the heterogeneity index (I^2^) was 81.9% (*p* < 0.001) and the pooled prevalence of disrespect and abuse of women during childbirth among women in East Africa was 46.85% (95% CI: 26.72, 66.97) (Figure 2).

### Subgroup analysis

**FIGURE 3:**
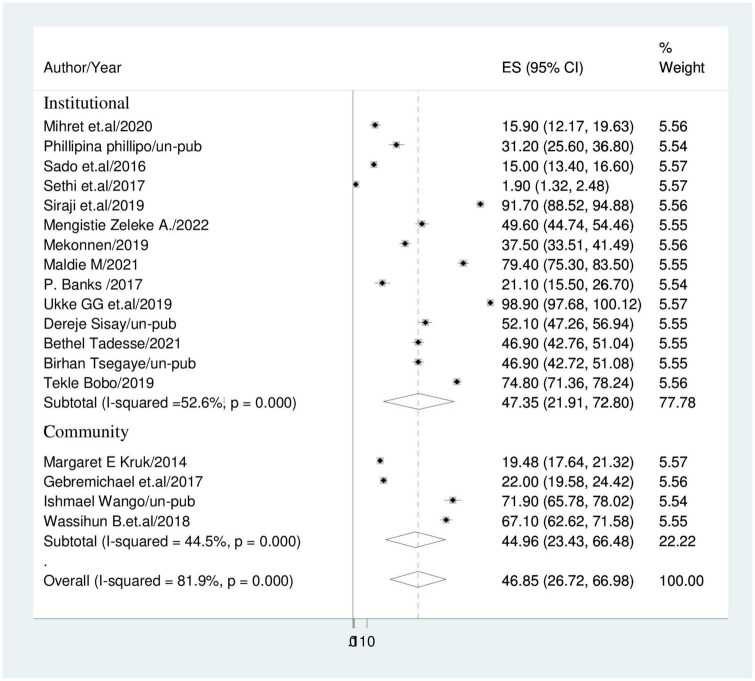
Forest plot displaying sub-group analysis based on study setting.

**FIGURE 4:**
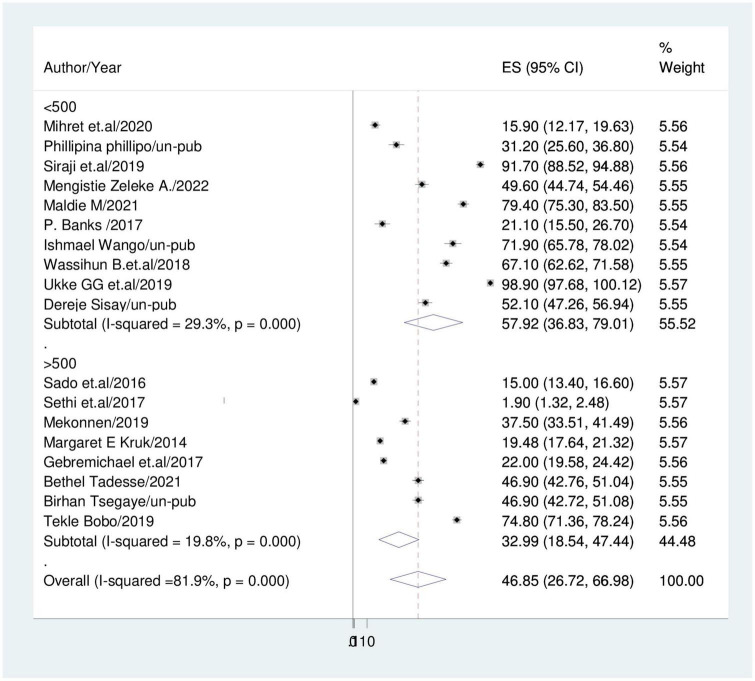
Forest plot displaying subgroup-analysis based on sample size.

Subgroup analysis based on sample size and study setting was carried out because this meta-analysis revealed a notable heterogeneity. Because of this, community based studies had the lower prevalence of disrespect and abuse during childbirth (44.96%; 95% CI: 23.43, 66.48); I^2^ = 44.5% than institutional based studies (47.35%; 95% CI: 21.91, 72.80); I^2^ = 52.6% (Figure 3). Studies with sample sizes of less than 500 had a prevalence of disrespect and abuse of women (57.92%; 95% CI: 36.83, 79.01); I^2^ = 29.3% while studies with sample sizes of more than 500 had a prevalence of (33%; 95% CI: 18.54, 47.44); I^2^ = 19.8% (Figure 4).

#### Heterogeneity and publication bias

We calculated a sub-group analysis based on sample size, and study setting to correct the reported heterogeneity of this study (I^2^ = 81.9%). Additionally, a univariate meta-regression utilizing the sample size and year as covariates was conducted to determine the root cause of heterogeneity. It demonstrated that the variability between research was unaffected by sample size or the year (Table 2).

**TABLE 2:** Meta-regression analysis of factors affecting between-study heterogeneity.

Heterogeneity source	Coefficient’s	Standard error	*p*-value
Sample size	4.29	3.07	0.21
Year	-63.74	68.41	0.95

**FIGURE 5:**
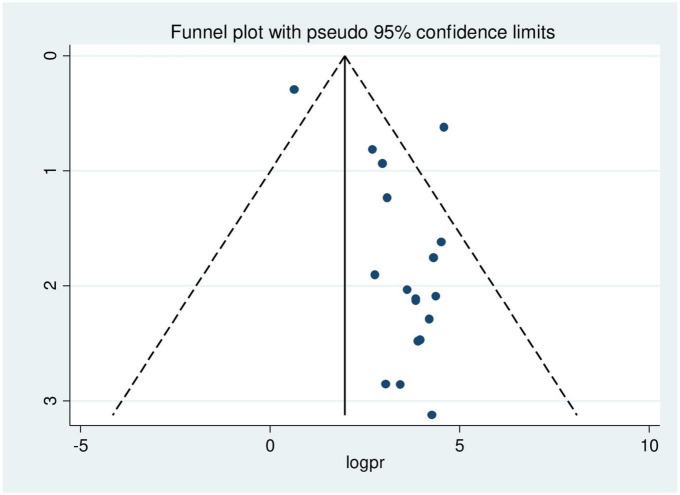
Funnel plot showing asymmetrical distribution of studies indicating the presence of publication bias.

**FIGURE 6:**
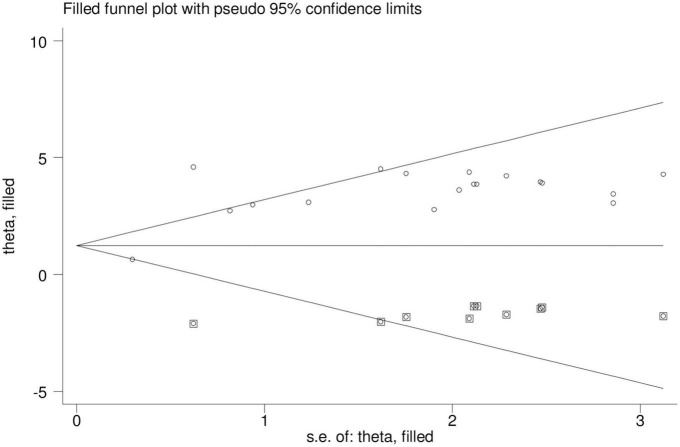
Trim and fill methods of analysis displaying the presence of eight missing studies causing for funnel plot asymmetry.

A funnel plot was used to analyze the presence of publication bias visually, while the Egger’s test and Begg’s test were used to assess it objectively. The funnel plot shows an unequal distribution of studies upon visual observation (Figure 5). The Egger test (*p* = 0.002) and Begg test (*p* = 0.041) suggested a significant publication bias, thus we conducted Duval and Tweedie trim-and-fill analysis to address it across the studies. The pooled prevalence of disrespect and abuse of women during facility childbirth was revised to 1.7% after the inclusion of eight studies within the fill and trim analysis. Therefore, trim fill analysis was employed to correct publication bias when eight papers were included in the funnel plot (Figure 6).

**FIGURE 7:**
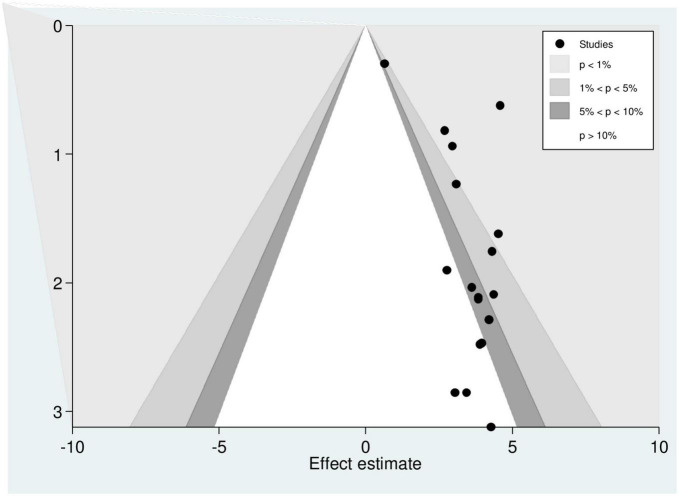
Counter-enhanced funnel plot showing that funnel plot asymmetry is due to the presence of publication bias.

We also did a counter-enhanced funnel plot to identify potential causes of the funnel plot’s asymmetry. Given that the majority of this area contains regions with statistically significance, publication bias is more likely to be the main cause of this funnel asymmetry (*P* < 0.01). Different shaded patches are present to show statistical significance. In particular, *p*-values higher than 0.10 correspond to the center white shaded area, *p*-values between 0.10 and 0.05 to the heavy gray shaded region, *p*-values between 0.05 and 0.01 to the medium gray shaded region, and *p*-values less than 0.01 to the area outside the funnel (Figure 7).

#### Leave –one-out-sensitivity analysis

A leave-one-out sensitivity analysis was carried out to detect the effect of each study on the overall prevalence of disrespect and abuse of women during childbirth by excluding one study at a time. In the sensitivity analysis, both Altahir et al. and Ukke et al. showed an impact on the pooled prevalence of disrespect and abuse of women during childbirth at health facility (Table 3).

**TABLE 3:** The pooled prevalence of disrespect and abuse of women during childbirth in East Africa when one study omitted from the analysis a step at a time.

Study omitted	Estimate	95% CI
Mihret et al. (41)	48.67	27.67–69.67
Phillipo (42)	47.77	26.93–68.61
Sando et al. (12)	48.73	26.33–71.12
Altahir et al. (51)	49.50	30.98–68.01
Siraji et al. (43)	44.21	23.84–64.57
Mengistie Zeleke and Melkie Bayeh (44)	46.69	25.84–67.53
Mekonnen et al. (45)	47.40	26.44–68.36
Kruk et al. (26)	48.46	26.47–70.46
Maldie et al. (46)	44.93	24.27–65.60
Gebremichael et al. (47)	48.31	26.87–69.75
Banks et al. (48)	48.36	27.52–69.20
Makumi et al. (49)	45.38	24.66–66.10
Wassihun et al. (50)	45.66	24.89–66.43
Ukke et al. (52)	43.77	29.95–57.58
Kebede et al. (53)	46.54	25.71–67.38
Tagesse et al. (54)	46.85	25.94–67.75
Negash et al. (55)	46.85	25.94–67.75
Tekle Bobo et al. (56)	45.20	24.50–65.91
Combined	46.85	26.72–66.98

### Factors associated with disrespect and abuse during childbirth

In this study, presence of complication during childbirth, receiving care at government hospitals, instrumental delivery, and poor wealth index were candidate variables of disrespect and abuse. Therefore, maternal disrespect and abuse were significantly associated with the presence of complications during childbirth, instrumental delivery, receiving of care at public hospitals and poor wealth index.

### Instrumental delivery

**FIGURE 8:**
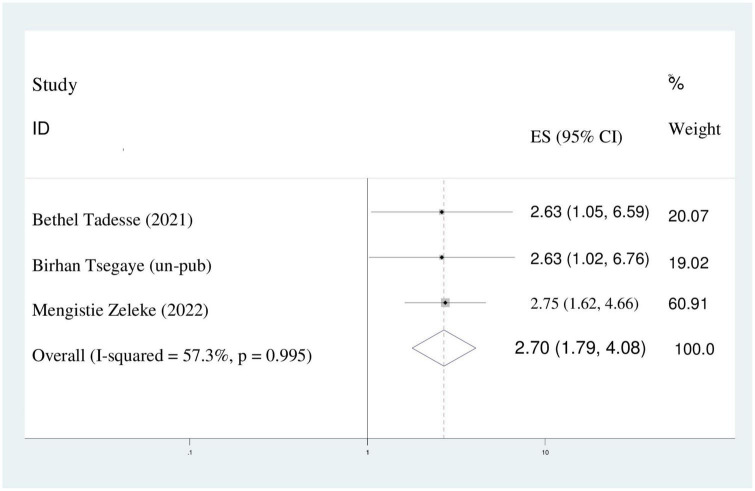
The pooled odds ratio displaying the association of instrumental delivery with disrespect and abuse of women.

According to this study, women who gave birth with instrumental delivery were 2.7 times more likely to be disrespected and abused as compared to women who gave birth with spontaneous vaginal delivery (stocktickerAOR = 2.70; 95%CI: 1.79,4.08). Random effect model was applied because of the existence of heterogeneity between studies (I^2^ = 57.3%) (Figure 8).

### Presence of complication during childbirth

**FIGURE 9:**
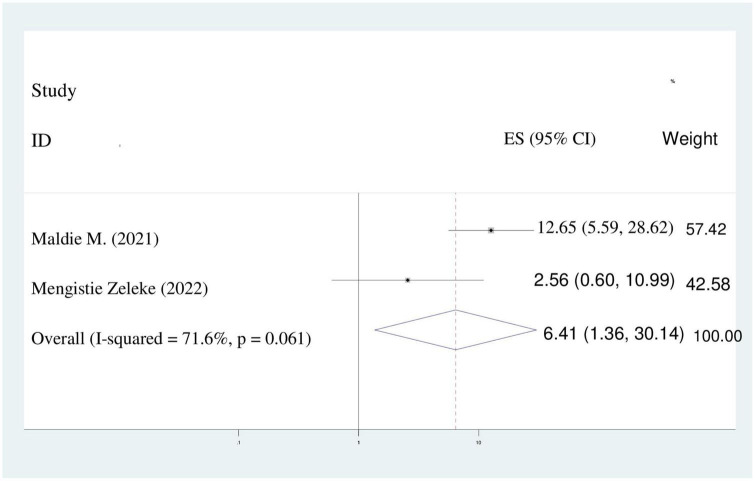
Pooled odds ratio displaying the association of presence of complications during childbirth with disrespect and abuse.

This meta-analysis found that women who had complications during childbirth were six times more likely to be disrespected and abused than women who didn’t have complication during childbirth (AOR = 6.41%;95%CI: 1.36–30.14). A random effect model was assumed because the value of I^2^ was 71.6% (Figure 9).

### Receiving of care at government hospitals

**FIGURE 10:**
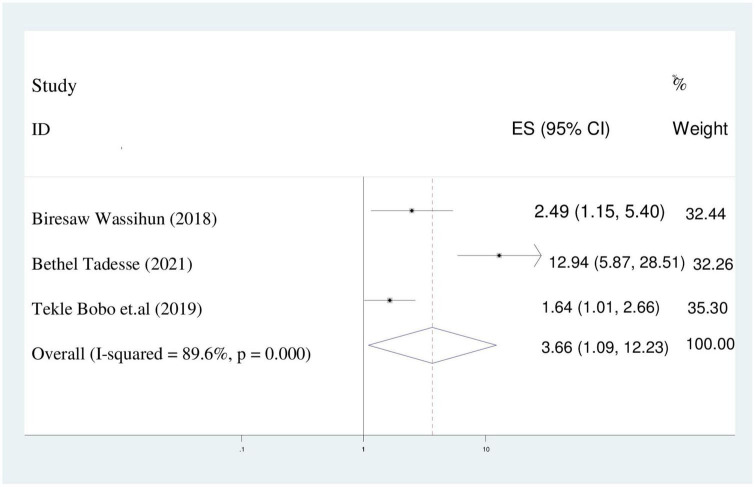
Pooled odds ratio displaying the association of receiving care at government hospitals with disrespect and abuse of women.

In this study, the odds of maternal disrespect and abuse during childbirth in government hospitals were 3.6 times higher than their counterparts (AOR = 3.66; 95%CI: 1.09–12.23). Because of I^2^ static indicated heterogeneity (89.6%), a random effect model was selected for the analysis (Figure 10).

#### Poor wealth index

**FIGURE 11:**
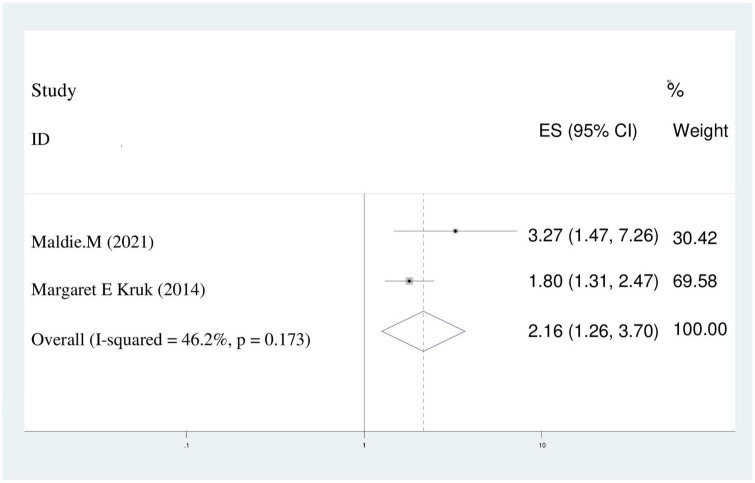
Pooled odds ratio displaying the association of poor wealth index with disrespect and abuse of women.

The chance of maternal disrespect and abuse of women who had poor wealth index were two times higher than women who had rich wealth index (AOR = 2.16; 95%CI: 1.26–3.70). We utilized random effect model because of the presence of heterogeneity (*I*^2^ = 46.2%) (Figure 11).

## Discussion

The fundamental rights of women, newborns, and families are violated when women are treated disrespectfully or violently when giving birth at a medical facility. One of the world’s biggest problems is the mistreatment of women who are giving birth in medical facilities. Due to the focus on reducing both maternal and newborn morbidity and mortality through the implementation of various measures such boosting institutional delivery and woman-friendly care. Meanwhile, using institutional delivery is difficult because of the disrespect and mistreatment of women while they are giving birth in a medical facility.

Three areas are highlighted in the World Health Organization’s (WHO) vision for the quality of maternal and newborn health under the experience component of quality of care: communication, emotional support, and dignity and respect (57). Dignity and respect place a premium on providing care that upholds women’s rights to privacy, confidentiality, and freedom from mistreatment such physical and verbal abuse and discrimination (58). In poor nations where a big proportion of clients are served by a small number of care professionals, disrespect and abuse are frequently more of an issue (58).

To improve the standard of care and the use of expert delivery services, respectful care during labor must be promoted (59). Abuse and disrespect have a negative impact on the use of competent delivery services, an intervention that has been shown to significantly lower maternal mortality across the globe (60). The key findings were that the estimate of disrespect and abuse of women during childbirth and disrespect and abuse of women were significantly related to the maternal wealth status, receiving at governmental hospitals, presence of complication during childbirth and instrumental delivery. The purpose of this systematic review and meta-analyses was to ascertain the general prevalence of disrespect and abuse among East African women as well as the factors that contribute to it. Therefore, the overall prevalence of disrespect and abuse of women during childbirth was 46.85% (95% CI: 26.72–66.98) in this study. The results of the current study are in line with meta-analysis studies conducted in Ethiopia (49.4%) (36) and Sub-Saharan African nations (44.09%) (61). This could be as a result of the research locations’ shared socioeconomic traits.

The results of this study are lower than those from studies done in Nepal (70.1%) (62), India (60%, 84.3%) (63, 64), Iran (75.5%) (65), Pakistan 97.4% (66), Pakistan 99.7% (67), and Peru 97.4% (68). The study population, the different health facility set up, and the definition of disrespect and abuse all may have an impact.

The results of this study was higher than those of studies conducted in Mexico (18.8%) (69), Brazil (18.3%) (70) and India (28%) (71) in comparison. Socioeconomic differences, variations in healthcare providers’ knowledge, attitudes, and skills, variations in healthcare facilities and systems, variations in study time and sampling methods, and differences in how disrespect and abuse of women during childbirth are defined at various healthcare facilities could all be contributing factors.

In thesis meta-analysis, presence of complication during childbirth, receiving care at governmental hospitals, poor wealth index, and instrumental delivery were predictors of maternal disrespect and abuse during childbirth. Women who utilized an instrument had a 2.7 higher likelihood of being disrespected and abused than those who gave delivery naturally vaginally. Studies from Pakistan (66) and India (64, 72) and their findings are in agreement with this one. The greater patient load and unfavorable patient-provider ratio in medical facilities may be the cause. The current study’s findings may be explained by the fact that, in comparison to higher-level settings, the delivery of health services in low-level settings is hampered by a lack of standards, leadership, supervision, and quality clinical care, as well as by a severe lack of multidisciplinary teams and training.

There was a 6-fold increase in the likelihood of disrespect and maltreatment among women who experienced complications during labor. This result agrees with the findings of the Indian study (72). She may have become susceptible to disrespect and abuse because of the risk of labor complications, which might have put the midwives on edge. Alternatively, the abuse or perceived disrespect may have been an effort by the midwives to shift the burden for the complications to her. There may be a statistically significant correlation between the lack of access to high-quality maternity care and disrespect and abuse of women. This is a crucial discovery since maternal stress of any kind slows down the labor process and raises the risk of problems (73). Therefore, in order to enhance maternal health outcomes, it is necessary to address the types of abusive provider behavior that can lead to difficulties.

According to this study, women with low wealth indices were twice as likely to experience abuse and contempt as women with high wealth indices. This result is in line with research conducted in Pakistan (74). The explanation could be because facility-based healthcare personnel treat wealthier women with greater respect and consideration than impoverished ones.

In contrast to women receiving care at private hospitals, the current study found that women receiving care at public hospitals were 3.7 times more likely to experience abuse and contempt.

The possible explanation for this is that, in contrast to health centers where there may be relatively fewer complicated cases and care providers are less stretched, hospitals with higher case loads and overflowing referrals of complicated cases that cause overcrowding may push care providers to provide abusive care.

In this study, a random-effect model was utilized to address a sizable variance that occurred in between-study heterogeneity. No single study significantly influenced the overall prevalence of disrespect and abuse of women, according to the results of a leave-one-out sensitivity analysis. To determine the presence of heterogeneity, sub-group analysis based on sample size, and study settings was conducted. The considerable heterogeneity may result from variations in the sample populations, variations in the paper’s properties, or variations in socio-cultural factor.

## Conclusion

To sum up, there was a significant frequency of abuse and disrespect toward women in East Africa after childbirth. The prevalence of disrespect and abuse of women during childbirth also varied depending on the study location and sample size. Significantly contributing factors to disrespect and abuse of women during childbirth included the presence of maternal difficulties during childbirth, low wealth index, receiving care at a government hospital, and instrumental delivery. It is advised that medical professionals adhere to the concepts of compassionate and respectful maternity care. Additionally, spontaneous vaginal birth and safe delivery practices have been recommended.

### Strength and limitations

This study has some limitations. First, the study protocol was not registered in the prospective international register of systematic reviews (PROSPERO). Second, articles were restricted to only being published in the English language. Third, all of the included studies were cross-sectional, which might affect the outcome variable because of other confounding factors.

This research has some strength. First, compressive electronic online international searching engines were used. Second, the predictors of disrespect and abuse were discovered.

## Data availability statement

The original contributions presented in this study are included in the article/Supplementary material, further inquiries can be directed to the corresponding author.

## Author contributions

NG conceptualized the study. NG and GA contributed to during data extraction and analysis. NG and KT wrote result interpretation. NG, GA, and KT prepared the first draft, contributed during the conceptualization and interpretation of results and substantial revision, and revised and finalized the final draft manuscript. All the authors read and approved the final version of the manuscript.

## Supplementary material

Supplementary Table 1 Prisma checklist.

Supplementary Table 2 Methodological quality assessment of included studies using Joanna Brigg’s Institute quality appraisal criteria scale (JBI). The eight item questions assessing inclusion criteria, study setting and participant, exposure measurement, objectives, confounder, statically analysis, outcome measurement and dealing confounder were used.

Supplementary Table 3 Risk of bias assessment for the included studies. The ten item questions of which four items assess external and six items assess internal validity were used.
