# Supplementary material for: Disrespect and abuse of women during childbirth at health facilities in Eastern Africa: systematic review and meta-analysis
Source: Front Med (Lausanne). 2023 Apr 20;10:1117116. doi: 10.3389/fmed.2023.1117116 (PMC10157168; doi:10.3389/fmed.2023.1117116)
Supplement: Supplementary Table 2 — Methodological quality assessment of included studies using Joanna Brigg’s Institute quality appraisal criteria scale (JBI). The eight item questions assessing inclusion criteria, study setting and participant, exposure measurement, objectives, confounder, statically analysis, outcome measurement and dealing confounder were used. [file Table_2.DOCX]

Table2. Quality assessment for the included Studies

| Item | Clearly defined inclusion | Describe study setting and participant | Valid and reliable exposure measurement | Objective and standard criteria for measurement | Identified confounder | Strategies to deal with confounders | Valid and reliable outcome measurement | Appropriate statically analysis | No of ‘yes’s ‘ |
| --- | --- | --- | --- | --- | --- | --- | --- | --- | --- |
| Mihret et.al | Yes | Yes | No | Yes | Yes | No | Yes | Yes | 6/8=75 |
| Phillipina Phillipo | Yes | Yes | Yes | Yes | No | No | Yes | Yes | 6/8=75 |
| Sado et.al | Yes | Yes | No | Yes | Yes | No | Yes | Yes | 6/8=75 |
| Sethi et.al | Yes | Yes | No | Yes | Yes | Yes | Yes | Yes | 7/8=87.5 |
| Mengistie Zeleke A | Yes | Yes | No | Yes | Yes | Yes | Yes | Yes | 7/8=87.5 |
| Mekonnen | Yes | Yes | Yes | Yes | Yes | No | Yes | Yes | 7/8=87.5 |
| Margaret E Kruk | Yes | Yes | No | Yes | Yes | Yes | Yes | Yes | 7/8=87.5 |
| Maldie M | Yes | Yes | Yes | Yes | No | No | Yes | Yes | 6/8=75 |
| M.W.Gebremichael et.al | Yes | Yes | No | Yes | Yes | No | Yes | Yes | 6/8=75 |
| P.Banks | Yes | Yes | Yes | Yes | Yes | No | Yes | Yes | 7/8=87.5 |
| Ishamel Wango | Yes | Yes | Yes | Yes | No | No | Yes | Yes | 6/8=75 |
| Wassihun B.et.al | Yes | Yes | No | Yes | Yes | Yes | Yes | Yes | 7/8=87.5 |
| Ukke GG et.al | Yes | Yes | Yes | Yes | No | No | Yes | Yes | 6/8=75 |
| Dereje Sisay | Yes | Yes | Yes | Yes | Yes | No | Yes | Yes | 7/8=87.5 |
| Bethel Tadesse | Yes | Yes | Yes | Yes | No | No | Yes | Yes | 6/8=75 |
| Birhan Tsegaye | Yes | Yes | Yes | Yes | Yes | No | Yes | Yes | 7/8=87.5 |
| Tekle Bobo | Yes | Yes | Yes | Yes | No | No | Yes | Yes | 6/8=75 |
