# Supplementary material for: Disrespect and abuse of women during childbirth at health facilities in Eastern Africa: systematic review and meta-analysis
Source: Front Med (Lausanne). 2023 Apr 20;10:1117116. doi: 10.3389/fmed.2023.1117116 (PMC10157168; doi:10.3389/fmed.2023.1117116)
Supplement: Supplementary Table 3 — Risk of bias assessment for the included studies. The ten item questions of which four items assess external and six items assess internal validity were used. [file Table_3.DOCX]

Table 3. Assessment of risk of bias for the included studies

| Item | External validity | | | | Internal validity | | | | | |  | |
| --- | --- | --- | --- | --- | --- | --- | --- | --- | --- | --- | --- | --- |
|  | Representativeness s of the target population | Representativeness s of the sampling frame | Radom sampling g or census | Minimal response e bias | Data were collected d directly | Acceptable e case definition used in the study | Valid and reliable measurement t | The same mode of data collection n for all study subject | Appropriate e length of prevalence period for parameter of interest | Appropriate numerators and denominator s of interest | No of yes | Summary of risk of bias |
| Mihret et.al | Yes | Yes | No | Yes | Yes | No | Yes | Yes | Yes | Yes | 8 | Low-  risk |
| Phillipina Phillipo | Yes | Yes | Yes | Yes | No | No | Yes | Yes | Yes | Yes | 8 | Low risk |
| Sado et.al | Yes | Yes | No | Yes | Yes | No | Yes | Yes | Yes | Yes | 8 | Low- risk |
| Sethi et.al | Yes | Yes | No | Yes | Yes | Yes | Yes | Yes | Yes | Yes | 9 | Low risk |
| Mengistie Zeleke A. | Yes | Yes | No | Yes | Yes | Yes | Yes | Yes | Yes | Yes | 9 | Low- risk |
| Mekonnen | Yes | Yes | Yes | Yes | Yes | No | Yes | Yes | Yes | Yes | 9 | Low- risk |
| Margaret E Kruk | Yes | Yes | No | Yes | Yes | Yes | Yes | Yes | Yes | Yes | 9 | Low- risk |
| Maldie M | Yes | Yes | Yes | Yes | No | No | Yes | Yes | Yes | Yes | 8 | Low- risk |
| M.W. Gebremichael et.al | Yes | Yes | No | Yes | Yes | Yes | Yes | No | Yes | Yes | 8 | Low- risk |
| P.Banks | Yes | Yes | Yes | Yes | Yes | No | Yes | Yes | Yes | Yes | 9 | Low- risk |
| Ishamel Wango | Yes | Yes | Yes | Yes | No | No | Yes | Yes | Yes | Yes | 8 | Low- risk |
| Wassihun B et.al | Yes | Yes | No | Yes | Yes | Yes | Yes | Yes | Yes | Yes | 9 | Low – risk |
| Ukke GG et.al | Yes | Yes | No | Yes | Yes | No | Yes | Yes | Yes | Yes | 8 | Low-  risk |
| Dereje Sisay | Yes | Yes | No | Yes | Yes | No | Yes | Yes | Yes | Yes | 8 | Low-  risk |
| Bethel Tadesse | Yes | Yes | No | Yes | Yes | Yes | Yes | Yes | Yes | Yes | 9 | Low- risk |
| Birhan Tsegaye | Yes | Yes | Yes | Yes | No | No | Yes | Yes | Yes | Yes | 8 | Low- risk |
| Tekle Bobo | Yes | Yes | No | Yes | Yes | Yes | Yes | Yes | Yes | Yes | 9 | Low- risk |
